# Commemorative Issue in Honor of Professor Paul J. Scheuer

**DOI:** 10.3390/md20110678

**Published:** 2022-10-28

**Authors:** Bill J. Baker, Mark T. Hamann, Yoichi Nakao

**Affiliations:** 1 Department of Chemistry, University of South Florida, 4202 E. Fowler Avenue, CHE 205, Tampa, FL 33620, USA; 2 Departments of Drug Discovery and Biomedical Sciences and Public Health, Colleges of Pharmacy and Medicine, Medical University of South Carolina, Charleston, SC 29425, USA; 3 Department of Chemistry and Biochemistry, Graduate School of Advanced Science and Engineering, Waseda University, Shinjuku-ku, Tokyo 169-8555, Japan

This Special Issue is dedicated to the memory of Professor Paul J. Scheuer (1915–2003), who was active at the University of Hawaii for more than fifty years. Paul is recognized as a pioneer, one of the first researchers to focus modern research technology (NMR, mass spectrometry) on the study of metabolites from organisms of marine origin. Trained in the 1940s in the laboratories of R.B. Woodward at Harvard University, and influenced by other faculty at Harvard with interests in the chemistry of nature, including Louis Fieser, Morris Kupchan and Gilbert Stork, Paul was well prepared to dive in, as it were, marine natural products. Not to discount his early work on endemic flora of the Hawaiian landscape, such as his studies of Kava Kava, but he soon learned of native Hawaiian uses of marine organisms for medicinal and other purposes. In the early 1960s, he traveled with his first postdoctoral scholar, Richard “Dick” Moore, who himself became an eminent natural products chemist and colleague of Paul’s, along a treacherous road on Maui to its end, in the community of Hana. Paul and Dick were in search of the “limu” (seaweed) famous among native Hawaiians for use as a spear-tip toxin. The seaweed was known to the Hawaiians as “limu make o Hana”, or the “deadly seaweed of Hana”. That “seaweed” turned out to be a previously undescribed zoanthid and was found to produce what we now know as palytoxin, one of the deadliest toxins. Paul made a number of other seminal contributions to marine toxins research, including studies of okadaic acid and ciguatoxin. Chemical ecology was his passion, and he took great pride in discoveries of the sequestration of metabolites among multiple trophic levels. He and his students discovered isocyanopupukeanane from nudibranchs and their sponge prey, ulapualide found in the Spanish Dancer nudibranch and its egg masses, and kahalalides in a green alga and its predatory sea hare. Many of Paul’s discoveries led to significant biomedical advances, most notably the kahalalides, which continue to advance in clinical trials as anticancer agents, but also including manoalide, the punaglandins, and halomon analogues. Paul took special delight in naming his compounds based on Hawaiian words, with the likes of popolohuanone and isocyanopupukeanane tripping the tongues of organic chemists ever since.

Paul was a consummate writer and editor, penning the first treatise on marine chemistry, titled simply enough “Chemistry of Marine Natural Products” [1]. He followed that with a series of edited books “Marine Natural Products: Chemical and Biological Perspectives” [2], which were the inspiration for many early chemists and biologists to enter the field. He ultimately authored over 300 papers, many describing first-in-class structural motifs, significant bioactivity and insights into biosynthesis, ecology, phylogeny, and pharmacology.

Working with Paul meant joining the Scheuer o’hana, or Scheuer family. Paul and his former students formed a network that continues to celebrate the successes and advancements of the o’hana. In recognition of their respect and gratitude, Paul’s former students, postdocs and visiting colleagues worked together in the early 1990s to establish the foremost award in our field, the Paul J. Scheuer award in Marine Natural Products.

We invite those who knew Paul, those who were inspired by him, and others who want to commemorate his life’s work to contribute to this Special Issue.
